# Justification of Social Egg Freezing and Regulatory Response: China’s Law and Practice

**DOI:** 10.1007/s11673-024-10409-0

**Published:** 2025-06-18

**Authors:** S. Gao, G. Liu

**Affiliations:** Law School, Southeast University, Nanjing, Jiangsu 211189 People’s Republic of China

**Keywords:** Bodily rights, Ethical dilemma, Individual autonomy, Regulatory system, Social egg freezing

## Abstract

Social egg freezing (SEF) refers to the act of a woman’s voluntary decision to preserve her eggs for future use. It is considered an expression of her right to bodily autonomy, allowing her to make decisions about her own reproductive capacity. As a form of exercising personal rights, SEF is aimed at preserving or extending fertility. Owing to the difference in attributes between it and the traditional “medical act,” SEF has sparked significant controversies in the academic world that remain unresolved. These debates stem from uncertainty and are often framed through the lens of consequentialist theory—focusing on potential social, ethical, or medical outcomes. For SEF, a more appropriate position should be taken, based on the theory of rights. This perspective centres on the individual’s legitimate claim to exercise bodily autonomy, particularly in relation to their reproductive potential. SEF is essentially a specific claim by a woman to exercise her right to control her own body as a means of realising her autonomy over her own eggs. To avoid the abuse of SEF, necessary legal regulations should be put in place.

## Introduction

Social egg freezing (SEF) is the term used when eggs or ovarian tissue are frozen for non-medical causes to be used later in life (Varlas et al. 2021), as opposed to medical egg freezing in the context of disease treatment. As its aim is to preserve fertility and provide eggs for future fertility, SEF is essentially non-medical egg freezing conducted by fertile and healthy women. There are differences in attributes between SEF and traditional medical treatments (Rimon-Zarfaty et al. 2021), generating ongoing debate and controversy in academic and ethical discussions. Divergences and controversies are inevitable in the discussion of freedoms and rights, especially in the matter of reproduction (Wang, Fan, and Shao 2024).

The first SEF lawsuit in China raises the theoretical inquiry: is the freedom to freeze eggs for social reasons justified? In the Chinese case, Xu Zaozao—an unmarried woman with a healthy reproductive system—sued the hospital after it denied her request to freeze her eggs, arguing that the hospital’s refusal violated her general personal rights. On July 22, 2022, the plaintiff lost the case (Chaoyang District People’s Court 2019). The court ruled that the hospital’s refusal to freeze the eggs was not illegal and did not infringe upon the plaintiff’s personal rights. Thus, the defendant does not have to bear the responsibility of tort. The case gives rise to the question of whether SEF should be permitted in China. The core of the argument concerned SEF is that it is doubtful whether can be regulated and guided within the existing legislative system. If the *Code of Assisted Human Reproductive Technology* (“ART Code”) does not apply to SEF, and SEF can only rely on the *Civil Code of the People’s Republic of China* (“Civil Code”) to find a basis for its rights, then this will lead to two questions: (1) What is the basis for the right to demand SEF? (2) If the medical sector rejects the request for SEF, even though SEF is legal, and the woman claiming egg freezing has that claim, then how will she claim the positive power of her right?

There are different views of “opposition,” “support,” and “neutrality” regarding SEF in China, with the controversy culminating in the first case of egg freezing. Opponents argue that SEF should not be legally recognized and cannot be a legitimate claim (Wang and Lv 2021). The proponents of SEF believe that individuals have a legitimate right to it and that its ethical risks are controllable (Hou and Yan 2022; Liu 2024; Zhang 2024). In addition, a lack of regulations for hypothetical risks cannot be a reason to limit the realization of an individual’s legal and legitimate rights (Wang and Lv 2021). The “neutrality theory” insists on a limited open attitude; that is, it affirms the guarantee of the right to SEF and at the same time emphasizes the social benefits of ethical constraints and legal regulation (Wang 2023). To a limited extent, it opens up SEF in a safe, effective, and controllable scenario. Controversy over SEF exists not only in China, but around the world, with supporting views (Goold and Savulescu 2009; Jones et al. 2018) and opposing views (De Sutter, Gerris, and Dhont 2008; Delvigne 2009; Petropanagos 2010; Baldwin et al. 2014). Moreover, views on the desirability and legitimacy of SEF vary from one country to another. It is viewed, in general, with caution. A few countries, including the United States, the United Kingdom, Japan, Spain, and Israel, have enacted legislation that is relatively inclusive and prudent, with a limited degree of liberalization of SEF. For instance, the United Kingdom (Human Fertilisation and Embryology Authority 2018), Germany, and the Netherlands (Rimon-Zarfaty et al. 2021) have permitted SEF, but public resources are limited to medical egg freezing. China has not yet liberalized SEF; furthermore, single women are explicitly forbidden from freezing their eggs (Ministry of Health [China] 2003).

In general, the reason SEF has triggered heated debates is obvious: SEF is inherently a non-disease treatment, a non-essential intervention. It has significant bioethical implications. On the one hand, SEF may raise a series of ethical problems, leading people to dismiss it as too risky; on the other hand, access to SEF is presented as a matter of individual rights, a matter of human dignity to be respected.

This paper does not deny that SEF can be a desirable freedom. The basic problem of SEF is in terms of ethical disputes, doctrinal interpretations, and legal regulations. Answering this question requires a detailed analysis of the right to SEF, including a breakdown of its legal characteristics. It also involves the construction of a dual legal framework: one that protects the individual’s right to SEF and another that regulates contracts related to the storage and custody of frozen eggs. In addition, acknowledging the risks associated with SEF is essential. Addressing these legal and ethical challenges can help shape appropriate regulations governing the actions of all parties involved in the SEF process.

## Critique of the Case Against SEF

In general, the dominant scholarly view opposing SEF in China is based on two main reasons: the impact of the ethical dimension and the lack of normative justification. In this paper, however, the justification is primarily consequential and non-deterministically consequentialist rather than presuppositional. These consequences, moreover, are only an indeterminate moral inquiry. The lack of normative justification cannot constitute a reason to oppose. The legislation itself lags behind, and “the law is not expressly stipulated” can be, in this regard, only applicable to the technical norms, without addressing the subject of the norms of the rights of the basis. As for the fear that SEF will lead to legal risk, the campaign should focus on making it illegal or unreasonable to engage in SEF rather than denying the individual the right to claim reasons for SEF. Therefore, neither of the above reasons constitutes a valid reason for opposing SEF.

### The Ethical Naturalist Opposition and its Critique

Although the right to SEF can satisfy the interests of the subject of the right, it also gives rise to ethical dilemmas. Admittedly, the “uncertainty” of SEF suggests that the negative impact of its goals refer exclusively to the social threats that may result in the formation of a certain degree of ethical risk, giving rise to the ethical naturalist opposition to SEF. Three main points comprise this opposition: the objectification of the subject, the impact on the family structure, and social injustice. Whether or not the various ethical risks that SEF may face inevitably lead to the lack of legitimacy of SEF remains unresolved.

#### The Core Rationale of the Ethical Naturalist Opposition

1. *Degradation of the subject* (Wang and Lv 2021). The subjectivity of human beings is generally believed to be the basis for the existence of human society, and is a principle that cannot be violated, giving rise to a set of moral ideals, norms, and beliefs. Therefore, SEF is an artificial manipulation of the human organism, which may intrude upon and erode human nature. Critics argue that extending the fertility period for women runs counter to human nature or disrupts the human condition in ways that merit ethical restraint (Bittner and Eichinger 2010). Two reasons underpin this assertion: the potential degradation of human dignity and the objectification of the body. In the case of human dignity, SEF is seen as a denial of natural reproductive limitations. It may be interpreted as an attempt to override the natural order. In addition, SEF is seen by some as causing women to misjudge their fertility and its outcomes, thereby creating a false freedom or behavioural fantasy that ignores the biological realities of aging and the limits of reproductive technology. In the case of the latter, SEF’s arbitrary disposal of women’s bodies makes frozen eggs a “commodity.” The separation of a woman’s eggs from her body and their physiological significance in the conception of life give them significant commercial value, making them vulnerable to being viewed as “commodities” to be traded, thereby degrading women’s dignity and equating them with reproductive tools.
2. *The “family shock” argument* (Shao 2023). If SEF is allowed, then everyone can delay childbearing in this way, which will directly lead to an increase in the age gap between parents and children (Wang and Guo 2018). The temporal gap between child and mother being too great, neither the mother nor the child would be able to give the other one the help they needed (Weber-Guskar 2018). At the same time, some women will be able to delay their marriage age without any restriction, which will result in them missing the best time to get married. This situation may have an impact on the family structure (Wang, Huang, and Liu 2015). The age difference between children and their parents will be large because of the advanced maternal age, which will make it impossible to fulfil the responsibility of maintenance and support. First, there will be obstacles to the child’s right to maintenance and support because of parental age; that is, parents may have the intention to maintain the child, but will be unable to do so. Second, there will be obstacles to the traditional reciprocal obligation of children to care for elderly parents. Parents may lose their ability to work due to ageing while their children are still minors. Additionally, single parenthood has become possible. Some take a conservative attitude toward single women giving birth, believing that childbearing is closely related to marriage. According to some, “single parenthood breaks the link between procreation and marriage and destroys marriage by the grace of God” (Qiu 2009, 25). At the root of this attitude is the wish to preserve the integrity of the family structure. From this point of view, SEF will have an impact on the traditional family structure, leading to the failure of family ethical constraints, the lack of family legal norms, and other difficulties.
3. *Exacerbating injustice* (Lei and Qiu 2023). The implementation of SEF depends, to a large extent, on the subject’s internal subjectivity and external conditions, which together influence the choice of SEF practices. Due to the high cost of the method, its selection remains a choice for only a few, reinforcing social inequality (Katsani et al. 2024). Once a difference appears in the external conditions of the subject, an imbalance will occur in the distribution of rights, which will, in turn, lead to social injustice. Currently, SEF is a form of interests exchange between equal social subjects, and it is unrealistic to ask the state to pay for it. In this way, only those who possess more resources enjoy the right to SEF, putting them in an advantageous position. Specifically, in the economic sense, they will have the right to control the timing of SEF. Conversely, those who lack the financial means to implement SEF will find that it becomes an “empty right.” In terms of SEF triggers, the subjects who choose SEF are often single women who have no energy or time to get married and have children because of work pressures. As a result, SEF does not fundamentally solve the anxiety of women who delay childbirth, but rather increases the unfairness of the technology among single women who do not have the financial means to do so.

### Rational Reassessment of the Ethical Naturalist Opposition

The above views may have some merit, but they cannot be taken as sufficient reasons to prohibit SEF. These views apply consequentialism or even utilitarianism to the issue of SEF. This is a type of “welfare theory” of risk; that is, it is impossible to avoid the ethical risks of SEF and its negative interpretation. However, this approach is not compatible with the logic of law, especially with the theory of individual rights.

1. *Reassessment of the rationale for subject derogation*. First of all, from the standpoint of human nature, SEF should be viewed a form of rational self-improvement. Human nature, in its essential form, encompasses the uniquely civilized and reflective capacities of human beings—such as foresight, autonomy, and long-term planning—which distinguish us from purely instinct-driven behaviour. As Chen and Wang put it, “human nature is the ability to choose freely, and this freedom of choice embodies the absolute responsibility of human beings for themselves” (2008). Hence, SEF itself should be seen as is a manifestation of the value of human nature —an act rooted in autonomy, foresight, and the rational planning of one’s life course. Second, the purpose of SEF is to preserve eggs for use at the time a woman chooses to procreate. Accordingly, the demand for SEF reflects a natural, deeply personal need among women who wish to become mothers, but who may face biological, social, or economic constraints. Third, to argue that SEF erodes human nature is to imply that it diminishes our level of civilization. Yet, the opposite is true. SEF exemplifies how humanity enhances its capabilities through technology—not to deny biology, but to manage and work with it more effectively.

The second counterargument is that SEF does not cause impairment of human dignity. “The human being, being endowed with reason, the capacity to judge things and the freedom to make decisions, has, unlike other animals, a supreme dignity” (Song 2016). Human dignity, depends on the autonomy and self-determination of the human being and the individual’s responsibility for their decisions. Therefore, SEF is not a denial of one’s own ability, but a choice to adjust one’s reproductive programme based on rational judgment. This enhances, one could say, the values of autonomy and justice. Women have more freedom concerning how to plan out their life if there is a longer time span during which they can have a child (Weber-Guskar 2018).The fact that people try to realize their own choices or freedom as much as possible within the scope of their own ability is itself a manifestation of human dignity. We do not agree with the implication that SEF is a technological manipulation of human autonomy. The key to the choice of SEF lies in the will of the subject, which is the autonomous choice of the rational economic person after weighing and balancing, which is the embodiment of the individual’s freedom. Oocyte cryopreservation offers women the chance to have genetic children later in life, reducing uncertainty and anxiety (Varlas et al. 2021). Allowing SEF behaviour is to provide people with more opportunities to choose, so that they are more free to choose their own judgement of the best time to have children.

Lastly, the argument that SEF may lead to the objectification of the body, although reasonable, does not apply to the premise that it may lead to the objectification of women’s bodies. If SEF leads to the objectification of women’s bodies, then the premise should be that SEF itself is illegal, but the fact is that SEF may lead to the objectification of women’s bodies only in cases of abuse. In the case of SEF, egg retrieval or freezing does not equate to the objectification of the body; according to the widely recognized right to bodily autonomy, an individual can reasonably dispose of the elements of their body. Not to mention SEF is the preservation of the function of the egg, the purpose of which is to realize the function of the body. The sperm, embryo, and other reproductive cells, which are homogeneous, can be separated from the body, and the same is true for the egg by analogy. There are exceptions to this phenomenon in the real world. For example, in the case of organ transplants, the human organ already exists as an object, but transplants are still permitted by society under the premise of strict regulations. Therefore, it is not desirable to prohibit SEF because it may lead to the objectification of the body.

(2) *Criticism of the family shock rationale*. SEF is argued to have caused women to delay childbearing and a surge in single births, leading respectively to a wider age gap between children and parents and changes in family structure. In this regard, three points will be made.

First, the wide age gap between children and parents is not an inevitable result of SEF. The argument that SEF may lead to wider intergenerational age gaps within socially integrated families is mainly based on two arguments. One, SEF transforms into a social phenomenon when it becomes possible for everyone to do it. However, SEF is only a precautionary behaviour in the event of a fertility crisis, not a preferred option. Women who plan to have children are generally aware of the risks of having children and will not delay their childbearing age indefinitely for their own safety. Even if SEF is fully liberalized, it will still be subject to the rationality of the subject. Two, when the family intergenerational age difference is too large, problems regarding the support and maintenance of the family itself are issues for family members to consider. Couples opting for egg freezing for fertility purposes will consider their own circumstances to make trade-offs. In addition, to prevent irrational behaviour by social groups, egg freezing can be strictly regulated in terms of procedure, and the age of the subject of egg freezing for reproduction can be appropriately restricted, taking into account medical conditions and safety assessments.

The second point is that Chinese family ethics is only against single parenthood, not the act of procreation, let alone women’s ability to enhance their fertility through SEF. There are two reasons for this: (1) First, SEF and single parenthood are different because SEF does not emphasize single parenthood but only enjoys autonomy in the act of procreation. In essence, SEF is intended to emphasize that women have reasonable control over their own eggs, whereas the scope of single parenthood is women’s reproductive rights after egg freezing. (2) As far as SEF is concerned, the idea of preserving women’s fertility is aimed at realizing the possibility of having children after marriage. In this regard, it does not contradict traditional Chinese family ethics, which emphasize the function of family reproduction. Therefore, some scholars advocate that “allowing single women to use assisted human reproductive technology is a safeguard for their reproductive autonomy, will not harm the interests of minors, and adapts to the current changes in reproductive policies and attitudes (Shi and Zeng 2022).” If this viewpoint is taken, then maternity benefits after SEF can also be used to realize the reproductive rights of single women.

The third point is that the adoption of SEF to prevent infertility will be socially beneficial. If women are not able to bear children, it may increase the instability of families and marriages and it will aggravate the ageing of the population. “The openness of fertility policy implies the acceptance of different forms of reproduction (Yang 2021).” In this sense, SEF can be regarded as a kind of “fertility support policy,” the essence of which is to maintain family stability and social harmony. At present, many factors hinder fertility, such as “not daring to have children, not being able to have children, not being allowed to have children, and not wanting to have children” (Chen and Sun 2022). SEF is intended to solve the problem of not being able to have children, which is not a cause of delaying the age of childbearing but a result of delaying the ages of marriage and childbearing. It is precisely because women of childbearing age are worried about missing the optimal age for childbearing that they choose SEF to alleviate their anxiety about not being able to have children. In this sense, SEF is not only an individual maternity insurance but also provides the potential for the families created by egg freezing to be prolonged, thus playing a stabilizing role for the children in the family.

(3) *Criticism of the rationale for social injustice.* The concept of social “fairness” implies two basic values: equality and justice. In the SEF scenario, equality is the premise and condition for social subjects to participate in social life, as well as the qualification for all citizens to have equal access to and enjoyment of rights. It can be said that equality is a universal value choice, which corresponds to the goal of “non-discrimination,” while SEF itself does not have the connotation of discrimination, and its implementation depends on the free choice of the subject, which is treated equally in the whole group of women who are eligible for egg freezing. For the unequal possession of resources that may be caused by SEF, social norms are required for the secondary distribution of resources. SEF itself is equal, but only due to the relative inequality caused by the scarcity of healthcare resources, which can be adjusted by using the social security system. As far as “justice” is concerned, in the SEF scenario, the inequality of distribution that may be arise due the differences of the subjects should not be attributed to the pressure from parallel groups caused by SEF but stems from the individual differences of the subjects themselves. In other words, even if it is not SEF but other healthcare measures, such confusion will still arise. The solution to this dilemma is not to eliminate the prohibition of SEF but to establish a fair procedure so that any principle agreed upon will be fair to every subject who voluntarily enters into the contract.

In sum, whether or not an action is justified is not determined by its consequences but by the characteristics inherent in the action itself. As for the technology of egg freezing itself, “Technique is a means to serve an end, and technology, unlike nature which contains its own principle of motion, is essentially an inert neutral instrument whose status depends on how one uses it, i.e., it requires a motivating cause” (Li 2024). Consequently, the key to the justification of SEF is its purpose (i.e., the question of the limits of that freedom). It is important to note that “freedom always comes with responsibility and risk; treatment and surgery are no exception.... The affected party may choose to accept them or to reject them” (Su 2008). Additionally, as far as the consequential analysis is concerned, it is clear that the harm of SEF has not yet occurred, and it will not necessarily lead to a harmful result that cannot be tolerated by the law. In terms of a purely naturalistic ethic, opposing the rights of the individual would be legally unacceptable. This means that the implementation of SEF depends fundamentally on the will of the person who freezes their eggs, which is justified by the protection of rights.

### The Legal Normativism Deficit View and its Identification

Supporters cite the “lack of legal normativism,” as demonstrated in the judgement of the “Xu Zaozao egg freezing case,” for two core reasons. First, SEF cannot be supported by China’s legislative norms (see table 1). According to the ART Code, SEF is unlawfully excluded. Second, SEF’s claim cannot be accommodated within the provisions of Article 990 of China’s Civil Code, “General Personality Rights.” Even if SEF’s claim is the embodiment of general personal rights, the refusal of medical institutions to freeze eggs is not unlawful. Note that the legality of SEF and SEF’s right to relief are two issues. On the basis of the above two reasons for making a decision, the former excludes the legality of SEF, while the latter is the choice of SEF’s right to relief. Therefore, the viewpoint that SEF is unlawful must be reviewed in the absence of legal norms.

**Table 1:** Legislation in China Relevant to SEF

Laws	2021	Civil Code of the People’s Republic of China	Effective
	2019	Basic Healthcare and Health Promotion Law of the People’s Republic of China	Effective
Administrative regulations	2001	Measures for the Administration of Assisted Reproductive Technology	Effective
	2022	Regulation on the Administration of Medical Institutions (2022 Revision)	Effective
Department specification	2001	Code of Assisted Human Reproductive Technology	Effective

### Xu Zaozao Egg Freezing Case

In the case of Xu Zaozao’s egg freezing, the court’s decision was made along the following lines: First, based on the *Measures for the Administration of Assisted Reproductive Technology* (“ART Administration Measures”) and the ART Code, it was held that “the egg freezing technology involved in this case … belongs to the category of assisted reproductive technology.” Second, under existing legislation, assisted human reproductive technology must be used for medical purposes. Third, according to the above norms, single women are not permitted to undergo assisted human reproductive technology. In addition, even if the egg freezing claim of the plaintiff is an object of personality right according to Article 990 of China’s Civil Code, the defendant did not violate the personality right of the plaintiff because of its unlawfulness. Indeed, there was nothing wrong with the outcome of the case. However, there is still room for discussion on its arguments.

On the one hand, while SEF is a medical act, it is not a human assistive technology. The legal definition of medical acts is generally considered to be the provisions of Article 88 of the *Detailed Rules for the Implementation of the Regulation on the Administration of Medical Institutions* (Revised), “Diagnostic and therapeutic activities: refers to the activities of making judgment on diseases, eliminating diseases, alleviating diseases, relieving pain, improving functions, prolonging life and helping patients to recover through a variety of checks and by using drugs and equipment, offering surgeries, and taking other methods.” Theoretically, medical acts can be broadly divided into acts of diagnosis, treatment, prevention of disease, and other medical acts according to their purposes, including medical cosmetology, helping or avoiding childbirth, organ transplantation, experimental treatment, and many other acts (Li 2023). Thus, SEF, as a form of egg retrieval and freezing with the help of medical means, can still be regarded as a kind of medical act. However, SEF is not a category of human-assisted reproductive technology. Article 24 of the ART Administration Measures defines the concept of “assisted human reproductive technology” and divides it into two types: artificial insemination and in vitro fertilization, including embryo transfer technology and its various derivatives. Assisted human reproductive technology refers to the use of medical technology and methods to artificially manipulate the gametes, haploids, and embryos to achieve the purpose of conception. SEF, on the other hand, does not refer to the act of “fertilization” but is limited to the act of egg retrieval and freezing. The act of “fertilization,” in comparison, is the act of using eggs. Therefore, it was difficult for the court to recognize SEF as an assisted human reproduction technique in this egg freezing case.

Did the medical institution’s refusal of SEF violate the duty of care?Regarding “duty of treatment,” Article 1221 of China’s Civil Code,^1^ Articles 27 of the *Law on Doctors of the People’s Republic of China*,^2^ Article 30 of the *Regulation on the Administration of Medical Institutions (2022 Revision)*,^3^ and other norms indicate that medical institutions, for the “emergency treatment” of patients, should provide timely assistance. Negative act of medical institutions is also a violation of the law.Although SEF is a preventive medical behaviour, it is not directed to the legal “treatment of disease.” In fact, medical behaviour consists of two kinds of legal relationships. One is the obligation of treatment, which refers to the “obligation of treatment corresponding to the level of medical care at that time,” that is, the medical institution’s inaction for the treatment of the disease. The other is the relationship of the medical service contract, which is a kind of autonomous relationship between the patient and the medical institution. Under the SEF scenario, the legal relationship between the egg freezer and the medical institution is a service contract relationship. Nevertheless, given that medical institutions are responsible for providing public health services, their willingness to enter into contractual agreements should be constrained by the relevant limitations. In cases of justified medical treatment that aligns with their level of medical care, medical institutions are obliged to take the necessary therapeutic measures in response to a patient's request for medical treatment, provided that it does not contravene the prohibitions set forth in the law. A women’s request for SEF from a qualified medical institution does not, in and of itself, contravene the prohibitions of the relevant legislation. Furthermore, the patient assumes all the adverse consequences of SEF based on the informed consent procedure, in accordance with the patient’s right to medical treatment. Patients are entitled to receive fundamental medical care, whereas physicians are obliged to provide treatment without undue refusal. As scholars have observed, physicians are obliged to provide patients with necessary and reasonable examinations and treatments within the scope of their professional duties. This implies that, irrespective of the severity of the condition, medical practitioners are obliged to provide treatment (Gan and Deng 2008). Legislation in countries around the world provides for the obligation of medical institutions to save lives in emergencies, which should be a mandatory provision. Violations of such provisions are not tolerated by law. In contrast, for non-emergency medical requests, medical institutions, as long as they have the appropriate medical level and ability, should provide treatment in accordance with professional ethics.

On the other hand, in terms of legislation, SEF itself is not illegal. According to the ruling of the case, SEF is an act prohibited by law, and is therefore illegal. The reason SEF is recognized as a violation of the law is that it is defined as human-assisted reproductive technology, yet Article 3 of the ART Administration Measures^4^ only considers the use such technology “for medical purposes” to be legal. Since SEF does not belong to human-assisted reproductive technology, so the judgment of the illegality of SEF cannot be carried out according to this logic. Regarding such judgment, there are actually no supporting norms in existing legislation. On the contrary, based on the egg freezer’s act of disposing of eggs, a legal basis can be found. According to Article 1003 of China’s Civil Code, “a natural person enjoys the right of inviolability and integrity of person. The physical integrity and freedom of movement of natural persons shall be protected by law. No organization or individual may infringe upon any other’s right of inviolability or integrity of person.” That is, “an individual’s right to consent to the autonomy of the body’s biological tissues” should not be infringed (Sun 2020). In addition to Article 1006 of the same law,^5^ an individual has the right to donate his or her bodily elements free of charge in accordance with the law.

In sum, the prohibition on single women under the ART Administration Measures, or the ART Code, is that they are permitted to use assisted reproductive technology, not that they are prohibited from medical claims of egg freezing. “Egg freezing is a contractual process of medical service between a woman and a reproductive assistance organization based on her autonomy and by agreement” (Wang and Wang 2021).

### Legal Consequences of the SEF Abuse Theory and its Rationalization

Another reason for the absence of legal normativism around SEF is the potential for legal and ethical issues, as highlighted by the SEF abuse theory (Zhang and Cai 2024). This theory argues that when laws do not clearly regulate SEF, it becomes vulnerable to misuse and should be approached with caution. SEF represents both extreme personal autonomy and a complete bypass of existing legal frameworks. If SEF becomes fully liberalized, two main outcomes are likely: using frozen eggs for personal reproduction and disposing of frozen eggs for other purposes. The second outcome can involve destroying the frozen eggs, donating them to others, or selling them commercially.

The commercial value of frozen eggs could encourage a market for egg trading, potentially leading to criminal behaviour and the need for legal intervention.While the person who freezes their eggs through SEF has the right to control what happens to them, current laws do not clearly define how frozen eggs should be handled. This situation is similar to existing legal debates about frozen embryos. These gaps highlight the urgent need for clearer legislation. When a person who has frozen their eggs loses autonomy or passes away, a legal and ethical question arises: can their parents or close relatives inherit the frozen eggs? This issue highlights broader concerns about the potential misuse of SEF.

Some argue that because SEF can lead to unregulated social problems, it should not be allowed. However, this conclusion can be reconsidered from two key perspectives:

1. SEF does not meet the conditions for a legal ban. SEF itself is not illegal, the risks associated with SEF are uncertain and not clearly defined, SEF remains within the bounds of what society can tolerate, and any legal restrictions must respect individual rights to freedom and privacy. Banning SEF purely based on potential risks would blur the line between law and morality, allowing moral concerns to overly influence legal decisions and interfere with private choices.
2. The law should prohibit misuse, not SEF. Regulation should establish clear boundaries and prevent problematic practices such as inheriting frozen eggs without clear consent, commercial sale of eggs, and other unauthorized uses.

These concerns should be addressed through specific laws that reflect the unique nature of SEF. Instead of banning SEF, legislation should regulate how it is used to prevent misuse while respecting personal freedoms.

## Normative Analysis of the Right to SEF

As noted earlier, the key to determining whether SEF is reasonable, legitimate, and legal lies in whether it is based on a legal right. If SEF creates a legal relationship, can it be defined as a “right”? Why does it matter if SEF is recognized as a right? And how are the rights and obligations of the people involved connected? The above questions are the core issues of this paper.

### Ethical Analysis of the Right to Social Egg Freezing

As far as the rights-based approach to SEF is concerned, it must be grounded in ethical principles. For SEF to be considered a legitimate right, it should strike a balance between individual freedom and social harmony. This means that SEF “must serve the human being, be of good value to the subject, be beneficial or harmless to the public interest of the society, and be socially acceptable and feasible” (Wang 2018).

First, as an ethical right, SEF supports the legitimacy of exercising personal rights. Ethical rights exist within interpersonal relationships and are shaped by both moral and ethical considerations. The ethical right to SEF reflects an interest-based relationship—specifically, the value placed on individual autonomy. This autonomy, or freedom of choice, is not only personally meaningful but also contributes to the broader social good.

The nature of rights has long been discussed, and according to the “voluntarist theory,” rights are rooted in individual autonomy—meaning individuals have the legal authority to make choices that must be respected. In this view, SEF is a valid expression of personal freedom grounded in ethical and legal reasoning. In this sense, rights and morality are interrelated; that is, “the will makes a man free, a free man is a moral man, and true free will is in accordance with reason” (Zhu and Du 2005). SEF, as a form of exercising personal autonomy, falls under the category of behavioural choice rights—it is an individual’s decision to control their own reproductive options.

If SEF is to be recognized as a right, it must meet two key criteria: one is complete autonomy, and the other is lack of harm to others. SEF is an act voluntarily done by a single woman and represents a form of protected freedom. While it may seem like a “passive” or reluctant choice due to social pressures, it still reflects the individual’s active decision to protect her reproductive options. Importantly, a woman’s choice to freeze her eggs does not impose burdens on others or on society—it does not harm others in any direct way.

SEF is, at its core, an expression of personal interest and autonomy. The relationship between the woman and the medical institution is a private, contractual one. If a medical provider were to be held responsible for the outcomes of SEF, it would require defining SEF as a medical treatment for disease—which clearly does not align with SEF’s purpose. Therefore, if a medical institution chooses not to provide SEF services, it should not be held liable for damages under tort law.

SEF does not inherently harm public welfare. Concerns about its potential to trigger moral decline or social risks are speculative and based on fears of misuse. These concerns fall under the “theory of consequences,” which focuses on what might happen if SEF is abused—not on the act itself.

Second, the right to SEF holds significant value for both individuals and society. For the individual, it supports personal dignity, autonomy, and the pursuit of well-being. For society, it reflects a broader concept of shared rights and the common good. SEF enables people—especially women—to protect their reproductive choices and pursue happiness through the use of medical technology. It represents a combination of personal interest, social progress, and scientific advancement.

At its core, SEF promotes reproductive autonomy and personal freedom. The decision to freeze eggs reflects not just a private choice, but also a contribution to the social fabric. Procreation plays an essential role in the structure and stability of society, and allowing individuals to rationally manage their reproductive futures is part of participating in the common good. However, individual freedom must still operate within certain boundaries to ensure it aligns with social values and the public interest.

There should be informed consent and equal contractual freedom: The relationship between the individual and the medical provider should be based on mutual consent and equal standing. Medical institutions are not liable for damages simply for declining to offer SEF, as long as the decision respects informed consent and legal guidelines. SEF should only be promoted within the boundaries of what society finds morally and ethically acceptable. It must not conflict with public values or exceed the limits of social tolerance. Governments should establish clear legal protections and behavioral guidelines to ensure SEF is practiced ethically and lawfully, in accordance with due process.

Third, the right to SEF reflects a new kind of social relationship—one that law and society are increasingly called upon to support. It is best understood as a liberty interest: the individual’s right to choose their own lifestyle, manage their fertility, and make decisions about their own body. As Wang (2023) puts it, “for natural persons, the elements of personhood, such as life, body, liberty, and health, are inherent and inseparable.”

According to Liu (2020), “the right to personal liberty is the freedom of a human being to dominate and determine their own body autonomously, and encompasses the full self-determination of the human being over their body.” Individuals have the legal right to make reasonable decisions about their own bodies, as seen in practices like assisted reproduction and organ donation. In reality, many women today face challenges such as delayed marriage, demanding careers, and limited partner options. These factors—especially among older, unmarried women—often lead to concerns about delayed childbearing or even infertility. In such cases, a woman has the right to take control of her fertility by freezing her eggs. SEF is a practical and legally manageable way for women to preserve their reproductive options. Its regulation is feasible in terms of defining its content, the conditions for making a request, and the available legal protections.

### Interpreting the Nature of the Right to Social Egg Freezing and Physical Rights

There is ongoing debate in legal theory about how to categorize the right to SEF. It has been variously described as “the right to freedom,” “the right to procreate,” “the right to body,” “the right to general personality,” and so forth. Because of these differing views, it remains important to clarify the legal nature of SEF rights. Defining their scope and structure helps avoid confusion in applying laws related to SEF.

This paper builds on existing legal scholarship to explore key questions about the legal foundation of SEF rights: (1) What is the ontology of SEF rights? (2) What specific rights are considered SEF rights?

The essence of the right to SEF is a universal right aimed at protecting the freedom of the individual. A specific right must be constituted by an act, which is understood as a fact, but to become a right, it must still be approved and supported by the people. The right to SEF, therefore, is expressed in the formal structure of “the act of freezing eggs (freedom)-social approval (recognition of value).” Ontologically, however, it is an act of freedom, with the core of the act being the freedom to dispose of it, which is expressed in the recognition of that freedom by the community at large. In other words, the right to SEF, that is, “A (the subject of the right) has the right to do φ(act),” does not require “a specific action by a specific obligor B” as a prerequisite for realizing the privilege (Chen 2014).In effect, this is a right of access to the world—meaning individuals have the freedom to act without interference from others. This right allows a person to exercise their autonomy, but it does not impose a duty on others to act on their behalf. In other words, while a person has the freedom to choose SEF, they cannot demand that others—such as medical providers—are legally required to help them carry it out.

No one has the right to obstruct someone else’s freedom to pursue SEF. However, this does not mean that medical institutions are obligated to provide the service. A clinic may choose not to perform egg freezing, but it cannot call for laws that prohibit it altogether.

Requiring a medical institution to perform SEF would only be justified if SEF were considered a medical treatment for a disease—which it is not. Rather, SEF is a voluntary action aimed at preserving fertility, and it falls under personal freedom. As such, it belongs within the broader category of the individual’s right to access the world and make autonomous choices without unjust interference.

From a legal perspective, the right to SEF is best understood as a bodily right. To define its legal attributes, we must (1) determine whether SEF is a public law right or a private law right, and (2) on the basis of determining the former, what specific type of right it should be. In this regard, this paper arrives at the following judgement.

First, this paper argues that SEF is a private law right, grounded in civil law. There are three main reasons supporting this view. (1) SEF rights and private law rights have the value of consistency. “The creation and granting of private rights is to enable civil subjects to enjoy in order to prevent and counteract the infringement from other civil subjects” (Liu 2012). If SEF were recognized only as a public law right, it would lack the legal tools necessary to protect individuals from violations by other private parties. (2) SEF involves the lawful and reasonable control of one’s own reproductive materials. Both parties involved—the individual and the medical provider—are equal civil subjects. Their interaction concerns the disposition of bodily elements, which clearly falls within the domain of private law. (3) As a private law right, SEF right can be included in the norms of China’s Civil Code. The Code provides a framework for defining how such rights are exercised, what their legal effects are, who holds them, how violations are addressed, and how remedies are applied.

Second, this paper argues that SEF is a claim to bodily rights, the essence of which is the protection of a woman’s right to control her own body. With advancements in life science and technology, the legal and ethical concept of the body has evolved to include not only the physical organism but also physical dignity. This broader understanding means the body is now seen as encompassing both material and spiritual interests. As Sun (2020) notes, human dignity is closely tied to both the right to the physical body and the right to the dignity of that body. Therefore, the protection of SEF rights is not only about reproductive freedom—it also reinforces the deeper value of human dignity, acknowledging both the personal and moral significance of control over one’s own body.

Regarding the civil right attribute of SEF rights, controversies abound in academic circles. First, there is the view that SEF rights fall under “general personality rights,” which can be covered by Article 990 (2) of China’s Civil Code. Article 990 includes a clause for “other personality interests,” which can reasonably be interpreted to cover SEF rights, given their aim of upholding human dignity.” Second is the view that sees SEF rights as part of the “right to personal freedom.” Since SEF allows individuals to make autonomous reproductive choices, it aligns with Article 1011, which protects individuals’ personal freedom under civil law. Third is the view that SEF rights are a form of reproductive autonomy, placing it within the broader category of the right to procreate. SEF is seen as a supportive method that enables individuals to plan when to have children, thus exercising their civil right to procreate. Fourth, there is the view of “independent right theory,” which holds that the right to SEF can be established separately as a specific new type of personality right (i.e., the “social egg freezing right”), which can be added to the specific personality right norms of China’s Civil Code. Fifth is the view that sees SEF as a component of of the “right to health,” as defined in Article 1004. According to this view, SEF enhances and preserves women’s reproductive health, thereby falling within the scope of health interests—which include both the proper development and function of bodily organs and the maintenance of normal physiological processes. Sixth is the view on the “right to body” theory, which holds that the right to SEF is essentially the free disposal of one’s own body elements.

Therefore, it is necessary to review the aforementioned views. First, concerning the “general personality rights,” while SEF may be interpreted under the umbrella of general personality rights (Article 990(2) of China’s Civil Code), this category is meant to support specific personality rights, not replace them. If SEF rights are better supported by a more specific right, that right should apply. Relying solely on general personality rights risks overlooking important legal details and weakening protections tied to human dignity. Second, the “right to personal freedom” misunderstands Article 1011, which protects a person’s freedom of physical movement—not control over their internal bodily elements like eggs. Thus, SEF falls outside the intended scope of this provision. Third, the “right to procreate” theory is problematic. The nature of this right, who holds it, and how it conflicts with other rights are still unclear. Because of this, we cannot assume that the right to procreate automatically includes the right to use SEF. While SEF appears to help delay childbirth and preserve future fertility, it is not directly tied to actually giving birth. SEF only involves freezing eggs, and there is no guarantee that childbirth will follow. If the subject of the right carries out the freezing of eggs for other purposes, or abandons the continuation of childbearing, then the act can be covered by the right to reproduce. Fourth, proposing SEF as a new independent right faces two challenges: (a) proving there is a legal gap that existing rights cannot fill, and (b) the high cost and complexity of creating a new legal category. If SEF can be reasonably interpreted within current legal frameworks, this is the more practical and effective path. Fifth, the “right to health” has an essential difference from SEF. SEF does not treat illness or restore health; it enhances reproductive options without involving any medical harm or deficiency. Therefore, it does not align with the legal definition of the right to health, which protects against threats to physical well-being and disease.

In contrast, the “right to body theory” is more accurate. This view is that SEF is an act of bodily autonomy: once an individual chooses to undergo egg freezing—a medical procedure involving surgical intervention—this choice, as long as it is lawful, should not be subject to interference by external forces. It is a voluntary act of self-determination and reasonable control over one’s body.

Traditionally, the right to the body has been understood as a negative right—a shield against violations of bodily integrity. However, it also contains a positive dimension: the right of a person to control and dispose of elements of their own body. In the case of SEF, the subject of the right is a single, fertile woman; the object is her eggs; and the effect is their preservation through freezing. This reflects a practical application of the broader legal concept of bodily rights.

With advancements in biotechnology, the right to the body has evolved to include a dominant power—the ability of individuals to actively make decisions about their own physical selves. SEF exemplifies this modern expansion of bodily rights, emphasizing the individual’s right to self-determination and reasonable control over her reproductive potential. It can be said that “frozen sperm and eggs [are the]… embodiment of the body’s functions and attributes … the value of personality, rather than the value of property …” (Liu 2020).

In practice, there are four main legal categories for how a person can dispose of or use their body: (1) disposal of blood and bodily fluids, (2) disposal of organs, (3) disposal related to medical procedures, and (4) disposal of accessory body parts like hair. Social egg freezing (SEF) falls into the third category—disposal related to medical procedures.

SEF is not an unrestricted right. It is subject to legal limitations, including who can undergo the procedure, under what conditions, and for what purposes. These restrictions form the legal framework governing SEF.

Importantly, while the law prohibits the commercial trade of germ cells (like eggs or sperm), it allows individuals to separate these cells from their bodies. This implies that SEF, as the act of collecting and preserving one’s own eggs for personal use, should be legally accepted—just as sperm donation is accepted, even when used by others.

The act of SEF contains two kinds of legal relationships: one is the medical contract based on egg extraction surgery, and the other is the custodial contract based on egg freezing and preservation. While both kinds comprise the act of disposing of bodily rights, in terms of legal relationship, the two have different rules. The former applies the rule of “informed consent,” which is the scope of medical self-determination, while the latter applies the rule of “invitation-commitment,” which is the scope of freedom of contractual expression.

Under Article 1219 of China’s Civil Code, the egg harvesting procedure requires the informed consent of the person undergoing it. This consent gives medical institutions legal protection from liability. If the consent does not meet legal requirements, the procedure may violate personal rights, and the medical provider could be held liable for a tort.

The rule of consent reflects an individual’s right to control medical decisions. This includes the right to be informed, to make decisions, to choose treatment options, and to accept the risks and responsibilities that come with those decisions.

In addition, after egg retrieval, the individual must enter into a valid egg custody agreement with the medical institution. This is governed by contract law and reflects the principle of freedom of contract. The custodian (medical institution) is then obligated to fulfil the terms of the agreement. However, in view of the special nature of the custody of frozen eggs, it is possible to formulate a standard contract for the custody of SEF with respect to the time of custody of frozen eggs, the activation of frozen eggs, and the disposal of frozen eggs. All these aim to adhere to the combination of independent contracting and record management, and the combination of protection of rights and interests and prevention of risks, so as to realize the whole process of regulation of the custody and utilization of frozen eggs.

## Ethics and Legal Regulation of the SEF

According to China’s Civil Code, individuals have the right to control their bodily elements. However, without proper legal regulations, the use of SEF may lead to serious risks—such as physical harm from the procedure, ethical concerns, and the illegal or improper use of frozen eggs. To support the responsible exercise of the right to SEF, it is essential to strengthen both ethical and legal frameworks. Reasonable limits should be placed on the freedom to use SEF, and a coordinated system of ethical and legal protections must be established and improved.

### Ethical Regulation of Social Egg Freezing

It is natural for a natural person who has not yet given birth and has the desire to do so to use SEF to preserve their fertility when it may be lost. However, SEF is prone to many unknown risks. To avoid these, it is important to uphold the standard of the common good, clarify the ethical principles guiding SEF, redefine the responsibilities of key parties, and strengthen the ethical direction of relevant policies.

First, the regulation must follow the standard of the common good. There is a sense in which “making a claim on......” is a “performative claim,” referring to an action that will have certain consequences (Zhu 2022). Similarly with SEF, there will be two effects: the fulfilment of individual rights and the impact on the public interest of society. A connection exists between SEF rights and the concept of the common good, and balance between them depends on how far the protection of SEF rights is extended. In a system based on the common good, protecting individual rights should not simply mean prioritizing personal freedoms—it should also consider the well-being of others and the broader interests of society. Therefore, in regulating SEF, ethical principles such as care and justice must be clearly defined. Individual rights should be exercised within reasonable limits, and the moral boundary between personal autonomy and the common good must be carefully maintained.

Second, the ethical principles of SEF should be clarified. *Opinions on Strengthening Ethical Governance of Science and Technology* outlines five core principles for the ethical governance of science and technology. These principles also apply directly to the governance of SEF.

Human-Centredness: SEF must respect and protect the rights and dignity of individuals. Practices that commodify eggs or objectify women are explicitly prohibited, as they violate personal dignity.

Respect for Life: Since SEF involves preserving reproductive potential, it is an expression of valuing life. To ensure safety, clear technical guidelines should be developed, along with strict standards for SEF practitioners and institutions. Access to SEF must be regulated to ensure safe and ethical practices.

Fairness and Justice: To avoid social pressure on certain groups to undergo SEF, public awareness must be guided responsibly. People should understand that SEF is not a universal solution to fertility issues and should make informed, voluntary choices. Additionally, the cost of SEF should be set based on the average economic capacity of social groups to ensure fair access to basic reproductive services. The state should also set up guidelines on the implementation of technical safety for practitioners and institutions. Furthermore, the state should provide relevant medical security facilities as much as possible and give full play to the secondary regulation function of social insurance benefits to promote social medical equity.

Prevention of Ethical Risks: In the process of SEF, it is necessary to uphold moral standards and protect human dignity. Individuals must be clearly informed about the purpose and implications of SEF, and their personal circumstances should be carefully reviewed to prevent illegal or inappropriate use of the procedure. Additionally, egg freezing institutions must strengthen their capabilities in key areas such as protecting personal privacy, ensuring safe storage of frozen eggs, and carefully managing the initiation of the egg freezing process.

Informed consent: Several key conditions must be met. SEF must be initiated solely based on the individual’s independent application. The egg freezing institution cannot unilaterally decide to perform the procedure. The individual undergoing SEF should have the ability to make decisions on their own. During the freezing and storage period, clear procedures must be established for renewing storage, initiating the use of frozen eggs, and determining final disposal. These steps must reflect and respect the ongoing consent and intent of the individual.

Third, the individual undergoing SEF must also take ethical responsibility for their choices. This responsibility can be understood in different ways—such as formal versus substantive responsibility or natural versus contractual responsibility. Therefore, when claiming the right to SEF, individuals should adhere to the concepts of ethical responsibility and natural responsibility and have a sense of responsibility for the eggs. These are the elements of their body with vital signs, as well as the social impact of their behaviour, that is, only to the extent that it is necessary for the preservation of one’s own reproductive capacity, and not to seek economic benefits. If, in the course of SEF, one’s subjective intent or negligence leads to illegal acts or damages, one should bear the resulting formal and contractual responsibilities and, in particular, should not transfer one’s responsibilities to the contractual counterpart, the egg freezing organization, to bear. As for the egg freezing organization, when it carries out the act of SEF, it must reasonably examine the feasibility, necessity, and legality of the act and fulfil the necessary duty of care and privacy protection obligation for the process of the act.

In addition to SEF actors, “the state, the government and other public authorities must be responsible for the pursuit of the overall interests of mankind” (Liu and Yi 2017); provide positive value guidance for the public to engage in SEF behaviours; require the public to focus on the subject of morality and self-discipline; and shape a self-control in line with the virtue of the “responsibility-consciousness” oriented SEF behaviour. It requires the public to pay attention to moral subjectivity and self-discipline, shape a self-control, virtue-oriented “responsibility” and “self-awareness” attitude of SEF value, and formulate and improve relevant policies and legislative norms to effectively regulate illegal behaviours.

### Legal Regulation of SEF

Needless to say, in the process of SEF, the improper exercise of rights will inevitably cause some legal problems. Thus, it is necessary to provide a legislative response to this, especially the procedural regulation of SEF claims. To ensure that egg freezers realize their right to SEF and prevent improper egg freezing, it is necessary to establish a standardized SEF implementation procedure and construct and improve the legal regulation system for SEF under the rule of law.

On the one hand, in the exercise of SEF rights, legislation must be enacted to clarify the boundaries of their rights. Article 1003, paragraph 2, of China’s Civil Code stipulates that no organization or individual may infringe upon the physical rights of others. In this way, it guarantees that “an individual’s behaviour will not be interfered by others, and he or she will be able to make choices in accordance with their own will without external constraints” (Gao and Li 2018). However, the legislation does not directly refer to unlawful interference with the right to the body, and its normative interpretation includes the prohibition of interference with the dominance of the body, which naturally encompasses the prohibition of interference with SEF. This requires that when an individual decides to carry out egg freezing, others may not interfere with its freedom; such an obligation is an obligation of omission, that is, as long as the subject of the obligation does not carry out acts that infringe on the right to the body of others, that is, the fulfilment of the legal obligation. In this regard, some scholars advocate that in the process of SEF, “the inaction of medical institutions cannot be defended from the prohibitions in departmental regulations and normative documents, and they are at fault for violating the statutory duty of care, which satisfies the element of culpability” (Wang 2023).In terms of the legitimacy of the right to SEF, the exercise of this right is grounded in the principle of bodily autonomy, specifically aimed at preserving a woman’s fertility. Fertility preservation must be the fundamental and legitimate purpose of SEF. Any act of egg freezing that is not intended for the purpose of reproduction should be explicitly prohibited, as it exceeds the permissible boundary of this right.

Furthermore, the use of frozen eggs must also be limited to the reproductive purposes of the woman who is the subject of the right. The eggs should not be used for unrelated purposes, such as research or commercial gain. Donation of frozen eggs may be permitted but must strictly follow legal provisions—particularly Article 1006 of China’s Civil Code—which requires informed and voluntary consent from the woman, compliance with formal procedures for donation, and a prohibition on the commercial sale or purchase of eggs.

In improving the content of the legislation, it is necessary to define the legal nature of frozen eggs as a special ethical object. In terms of its ownership and disposal, the subject of the right can only be limited to the person who freezes the eggs, while in terms of the preservation and supervision of the frozen eggs, it should be limited to the egg freezing institution that carries out the act of SEF. The frozen eggs should not be disposed of arbitrarily without the consent of the woman and a proper procedural review. However, in practice, situations may arise where the legal status of the frozen eggs becomes unclear—for instance, due to the death of the subject, failure to renew the storage agreement, or the subject’s loss of contact with the medical facility. Currently, Chinese law does not provide specific regulations on how such eggs should be disposed of.

Therefore, it is essential that egg preservation is governed by a clear, legally binding agreement made in accordance with existing laws. This agreement must outline who has custody of the frozen eggs and how they should be handled under different circumstances. Improper disposal—such as violating the agreement or legal requirements—can result in criminal, administrative, or civil liability for infringing upon the subject’s legal rights. For example, if a custodian unlawfully sells the frozen eggs without the subject’s consent, they may be subject to criminal penalties in accordance with Article 234-1, paragraph 1 of the *Criminal Law of the People’s Republic of China*, “Organized Selling of Human Organs,” and shall be held civilly liable in accordance with the provisions of Article 1007 of China’s Civil Code and the custodianship agreement of the frozen eggs.

In addition, a cautious attitude must be adopted on whether frozen eggs can be inherited for two reasons. First, recognizing the inheritance of frozen eggs undermines the original intent of self-elective egg freezing (SEF). Once the subject of the right is no longer alive, their autonomy—and the potential to personally exercise reproductive rights—ceases. Therefore, the reproductive purpose of the frozen eggs can no longer be fulfilled by the rightful subject, and their value cannot be ethically or legally transferred to others. Second, China maintains a firm prohibition against illegal surrogacy. Allowing the inheritance of frozen eggs could create legal loopholes that indirectly encourage or facilitate surrogacy arrangements, which are contrary to current law and public policy. As a result, the interpretation of rights related to SEF must remain narrowly defined. The principle of limited autonomy must be upheld—meaning the subject’s rights over their frozen eggs should be exercised only within clear legal boundaries and not extended beyond death or into third-party use.

On the other hand, in the SEF process, legislation is required to regulate its procedures. The inquiry of what SEF should do should be focused on institutional norms. The legal system for behavioural norms is generally believed to consist mainly of “soft law” principles and guidelines and “hard law” legal regulations. However, for SEF, there are only “soft law” indirect regulations, such as the ART Administration Measures, *Measures for the Administration of Human Sperm Banks*, *Guiding Principles for the Application of Assisted Human Reproductive Technology (2021),* and the *Opinions on Strengthening Ethical Governance of Science and Technology*. There are no “hard laws” directly regulating SEF (i.e., there is no separate legislative document in the legal sense).

Since SEF is a claim of bodily rights, it can be adjusted by China’s Civil Code, but it is only for the protection of rights, and the code still lacks procedural norms for the exercise of rights. Therefore, to properly regulate SEF, and to safeguard the exercise of related rights, laws governing gene technology, assisted reproductive technology, and organ transplantation should be established or expanded. These legal frameworks should create a robust procedural guarantee system for SEF, emphasizing both rights protection and institutional accountability.

First, strict examination and evaluation procedures must be established. Before any SEF procedure begins, egg-freezing organizations must implement comprehensive evaluation processes. Applicants must undergo a medical and psychological assessment to ensure they meet eligibility criteria. This includes an evaluation of the individual’s health status, reproductive intentions, and basic personal circumstances. Organizations must also provide full disclosure of the risks and potential adverse effects of the egg freezing process. Only upon meeting these requirements can an applicant proceed to sign an egg freezing contract. This contract must explicitly outline the responsibilities for egg storage, usage restrictions, procedures for initiating egg use, and the time limit for storage.

Second, rules must be established for the separation of examination and evaluation from the act of “freezing and storage.” To prevent misuse and reduce risk, the stages of SEF—evaluation, freezing, storage, and disposal—should be functionally separated. Different teams or personnel should be responsible for each stage. This separation increases transparency, minimizes conflicts of interest, and reinforces accountability. It also elevates the professional standards expected of SEF practitioners, ensuring that each phase is handled with appropriate care and expertise.

Third, egg freezing storage protection procedures must be established. During the storage period, institutions must actively monitor the condition of both the frozen eggs and the legal status of the subjects. This includes maintaining a clear duty of care to preserve the eggs safely and effectively. The subject has a continuing right to know, meaning they must be able to access status reports on their frozen eggs at any time. In the event of loss, degradation, or other emergencies, institutions are required to notify the subject promptly.

Fourth, an ex post facto supervision programme must be established. Once an SEF contract is activated, designated personnel must oversee compliance with legal, contractual, and ethical obligations. This includes monitoring the storage conditions, managing contractual terms, and reporting any changes or incidents. These records should be submitted to relevant regulatory bodies for review and supervision.

## Conclusion

Along with the change in China’s reproductive policy, societal attitudes toward SEF have emerged as an issue that cannot be ignored. Advocates of SEF often view it as a form of reproductive “insurance”—a proactive measure to preserve fertility, rather than an endorsement of assisted reproduction. Protecting the right to SEF, therefore, not only affirms an individual’s bodily autonomy but also aligns with the broader objectives of China’s current fertility strategy.

Despite the ethical dilemmas SEF may present, effective regulation is possible through the reshaping of ethical norms and the advancement of legal standards. Legislation plays a key role in this process, particularly through the development of a framework that permits limited openness—where SEF is allowed under clearly defined conditions. Over time, these specific rules contribute to the establishment of a coherent and comprehensive bioethics legal system.

To ensure meaningful protection of SEF rights, it is essential to clarify and balance the rights and responsibilities of all stakeholders: the individual, medical institutions, family members, and society at large. Such a multidimensional approach can uphold individual autonomy while promoting ethical practice and safeguarding collective social interests. In doing so, a harmonious balance between bioethics, rights protection, and public policy can be achieved.

## Footnotes

1 Article 1221 of China’s Civil Code stipulates that “Where any medical staff member fails to fulfill the obligations of diagnosis and treatment up to the standard at the time of the diagnosis and treatment and causes any harm to a patient, the medical institution shall assume the compensatory liability.”

2 Article 27 of the *Law on Doctors of the People’s Republic of China* stipulates that “For patients in need of emergency treatment, doctors shall take emergency measures for diagnosis and treatment, and shall not refuse to give first aid treatment. … Doctors who cause damage to the recipient as a result of voluntarily administering first aid are not liable for civil liability.”

3 Article 30 the *Regulation on the Administration of Medical Institutions (2022 Revision)*, “medical institutions shall immediately resuscitate critically ill patients. Patients that are unable to receive diagnosis or treatment due to restriction of equipment or technical conditions shall be transferred in a timely manner. ”

4 Article 3 of the ART Administration Measures stipulates that “the application of assisted human reproduction technology shall be carried out in medical institutions for medical purposes and in accordance with national family planning policies, ethical principles and relevant legal provisions. It is prohibited to buy or sell gametes, congeners or embryos in any form. Medical institutions and medical personnel shall not carry out any form of surrogacy techniques.”

5 Article 1006, paragraph 1, of China’s Civil Code stipulates that “A person with full capacity for civil conduct has the autonomy to decide to donate his human cells, human tissues, human organs, and remains in accordance with the law. No organization or individual may force or induce any person to make donation or deceive any person into making donation.”
